# Inhibitory KIR–HLA receptor–ligand mismatch in autologous haematopoietic stem cell transplantation for solid tumour and lymphoma

**DOI:** 10.1038/sj.bjc.6603913

**Published:** 2007-07-31

**Authors:** W Leung, R Handgretinger, R Iyengar, V Turner, M S Holladay, G A Hale

**Affiliations:** 1 Department of Hematology-Oncology, St Jude Children's Research Hospital, Memphis, TN, USA; 2 Department of Pathology, St Jude Children's Research Hospital, Memphis, TN, USA; 3 Department of Pediatrics, University of Tennessee Health Science Center, Memphis, TN, USA

**Keywords:** natural killer cells, solid tumour, lymphoma, autologous transplantation

## Abstract

Genes that encode killer Ig-like receptors (KIRs) and their HLA class I ligands segregate independently; thus, some individuals may express an inhibitory KIR gene but not its cognate ligand. We hypothesised that these patients with KIR–HLA receptor–ligand mismatch have a low risk of relapse after an autologous haematopoietic stem cell transplantation (HCT). Sixteen consecutive patients with lymphoma or solid tumour were enrolled onto a prospective study. They received high-dose busulphan and melphalan followed by autologous CD133^+^ HCT. We found that 8 of the 16 patients experienced disease progression after autologous HCT, including 5 of the 6 patients (83%) with no inhibitory KIR–HLA mismatch and 3 of the 6 patients (50%) with 1 mismatched pair; none of the 4 (0%) patients with 2 mismatched pairs experienced disease progression. Survival analyses showed that inhibitory KIR–HLA mismatch was the only significant prognostic factor (*P*=0.01). The potential applicability of the receptor–ligand mismatch model to autologous HCTs and to patients with lymphoma or solid tumour is clinically significant because of the prevalence of the HCT procedure.

Human NK cells are regulated by killer Ig-like receptors (KIRs) that recognise specific groups of HLA class I alleles (Lanier, 2005). Both KIRs and their HLA ligands exhibit tremendous population diversity, and the KIR genes on chromosome 19 and HLA genes on chromosome 6 segregate independently (Hsu *et al*, 2002; Parham, 2005). Thus, many individuals have an inhibitory KIR gene but not its corresponding ligand, while others may have a certain KIR ligand but not its corresponding KIR (Leung *et al*, 2004; Parham, 2005).

In corroboration with these genetic findings, we found that the risk of relapse after haploidentical haematopoietic stem cell transplantation (HCT) in patients with haematologic malignancies was best predicted by a model taking into consideration the expression of inhibitory KIRs on the donor NK cells and the absence of corresponding KIR ligands in the recipient's HLA repertoire (a KIR–HLA receptor–ligand mismatch model) (Leung *et al*, 2004). For those HCTs in which neither the donor nor the recipient had the cognate ligand for the donor's inhibitory KIRs, the patients were found to be at low risk of relapse. Indeed, autoreactive NK cell clones, which are KIR2DL2/2DL3^+^, 2DL1^−^, and 3DL1^−^, have been found in healthy donors who lack HLA-C^Asn80^ (the ligand for KIR2DL2/2DL3), and NK cells from approximately 50% of these individuals were cytotoxic against autologous CD34^+^ cells (Grau *et al*, 2004). Therefore, it is logical to examine the application of the receptor–ligand mismatch model to autologous HCTs. Because lymphoid leukaemias and solid tumours such as neuroblastoma are susceptible to KIR–HLA mismatched NK cell–mediated lysis (Leung *et al*, 2004, 2005a), we conducted a prospective study to test the hypothesis that patients with lymphoma or other solid tumour who receive autologous HCT have a low risk of relapse if KIR–HLA mismatch(es) is present.

## PATIENTS AND METHODS

### Autologous HCT

All patients were enrolled onto an autologous HCT protocol approved by the Institutional Review Board, and the patients or their legal guardians gave the written informed consent. After enrolment, all patients received 10 mcg kg^−1^ per day G-CSF for 5 days and then underwent leukapheresis for peripheral blood stem cell collection. Apheresis products were selected for CD133^+^ cells by using the CliniMACS system for tumour-cell purging, except for two that had low initial cell count. Conditioning consisted of busulfan (37.5 mg m^−2^ per dose every 6 h intravenously for 16 doses) and melphalan (70 mg m^−2^ per day for 2 days). After stem cell infusion, all patients received G-CSF, starting 5 days after HCT until ANC was >3000 mm^−3^ for two consecutive days.

### HLA and KIR typing

All samples were HLA typed by DNA methods as previously described (Leung *et al*, 2004). KIR genotyping was performed by using a genotyping kit from Pel-Freez (now Dynal Biotech, Invitrogen, Carlsbad, CA, USA) and KIR phenotyping was determined by flow cytometry analysis, as some of the KIRs may not be expressed on the cell surface (Leung *et al*, 2005b). Thus, a KIR was classified to be positive for a particular patient only if that KIR gene was tested positive by genotyping and was also found to be expressed by NK cells in phenotyping. The natural cytotoxicity of NK cells against K562 cells was determined by a standard europium release assay with a 40 : 1 (E : T) cell ratio (Leung *et al*, 2004).

### Statistical analysis

The cohort was divided into two risk groups based on the inhibitory KIR–HLA receptor–ligand model and cytotoxicity model, which have been described previously (Leung *et al*, 2004). Briefly, a patient was classified as being at low risk by using the KIR–HLA receptor–ligand model if at least one of the inhibitory KIR genes expressed on the patient's NK cells did not recognise any of the HLA molecules in the patient's HLA ligand repertoire. The patients in the low-risk group were then further categorised by the number of receptor–ligand mismatch pairs. The hypothesis was that a larger number of KIR–HLA mismatch pairs would result in greater antitumour activity (Leung *et al*, 2004). The cytotoxicity model measured the general cytotoxicity of engrafting NK cells against K562 cells (Leung *et al*, 2004). The Perugia's KIR ligand incompatibility model was not used, because all grafts were ligand matched with the recipients by definition (ie, the KIR ligands present in the graft were identical to those in the patient in all autologous HCTs). Event-free survivals (EFSs) were estimated and compared by using the method of Kaplan–Meier and log-rank statistics. Event-free survival was defined as the time from transplantation to disease progression or death, whichever occurred first. Because there was no transplant-related mortality, the EFSs were identical to progression-free survivals and there was no competing event in the evaluation of the cumulative incidences of disease progression. Cox's regression was used to assess hazard functions. Covariates included receptor–ligand mismatch, type of malignancy, disease status at the time of HCT, number of CD133^+^ cells in the graft, and natural cytotoxicity. Survivors were censored at a cut-off of 1 October 2006.

## RESULTS

**Figure 1:**
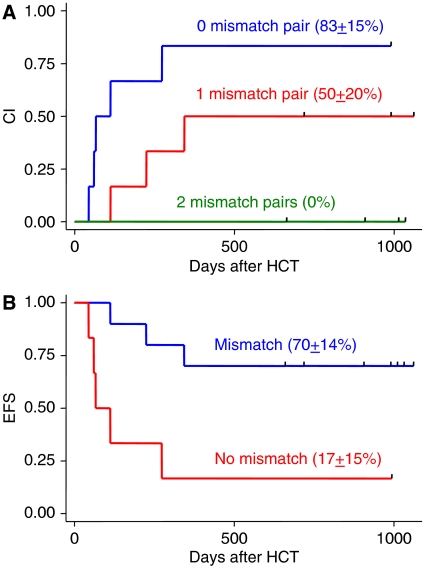
Significance of inhibitory KIR–HLA receptor–ligand mismatch. (**A**) Cumulative incidence (CI) of disease progression in patients with different numbers of receptor–ligand mismatch pairs (*P*=0.02). (**B**) Event-free survival (EFS) of patients with receptor–ligand mismatch and of those with no mismatch (*P*=0.01). Progression-free survival was identical to EFS because there was no transplant-related mortality.

Sixteen consecutive patients in a prospective study underwent autologous HCT. There was no transplant-related mortality (Table 1). Eight patients experienced disease progression after autologous HCT, including five of the six (83%) patients with no inhibitory KIR–HLA mismatch and three of the six (50%) patients with one mismatched pair; none of the four (0%) patients with two mismatched pairs experienced disease progression (Figure 1A). Survival analyses showed that KIR–HLA mismatch was the only significant prognostic factor (Figure 1B). The type and status of malignancy at the time of HCT, the number of CD133^+^ cells in the graft, and the cytotoxicity model measuring the lysis of K562 cells by engrafting NK cells were not significant prognostic factors (Table 2). The median time to relapse for the patients in the high-risk category with no inhibitory KIR–HLA mismatch was only 89 days (Figure 1B), whereas that for patients in the low-risk group with mismatch has not yet been reached after a median follow-up of 812 days.

This cohort provides a unique opportunity not only to investigate the prognostic significance of the KIR–HLA receptor–ligand model, but also to examine the KIR ligand repertoires of patients with solid malignancy, because HLA typing is typically not performed for autologous HCTs. We found that five patients in this cohort were Cw7 homozygous, and four other patients were heterozygous for Cw7. The frequency of Cw7 homozygosity observed in this cohort was 31.3% (95% confidence limits, 11.0 and 58.7%), which was significantly higher than that predicted by using data from the general population adjusted for ethnic group (8.6%; *P*=0.009 by binomial exact test) (Allele Frequencies in Worldwide Populations Website, 2006). Cw7 is a member of the HLA-C^Asn80^ allotypes. Cancer susceptibility in individuals homozygous for HLA-C^Asn80^, as well as protective effect by HLA-C^Lys80^, was observed in cervical cancer and melanoma (Carrington *et al*, 2005; Naumova *et al*, 2005).

## DISCUSSION

NK cells in our patients after autologous HCT were primarily derived from stem/progenitor cells, because mature NK cells were depleted *in vivo* and *ex vivo* by the myeloablative conditioning and CD133^+^ cell–selection procedure. During the period of receptor acquisition, subsets of NK cells may express a certain inhibitory KIR with no cognate ligand. Recent studies in healthy mice have demonstrated that NK cells expressing inhibitory receptors with no self-ligands exist (Fernandez *et al*, 2005; Kim *et al*, 2005). These cells are hyporesponsive in steady state, because they have not undergone the ‘licensing’ process (Kim *et al*, 2005; MacDonald, 2005). However, the hyporesponsive state of these ‘unlicensed’ NK cells is not permanent, as they can be activated easily in response to proinflammatory cytokines that activate almost all NK cells during infection (Biron *et al*, 1999; Kim *et al*, 2005). Thus, the licensing effect was much less prominent among preactivated NK cells, a finding suggesting that the licensing requirement could be circumvented in specific situations (Kim *et al*, 2005). The high-dose cytotoxic chemotherapy given to our patients may provide the proinflammatory setting that favours autoreactive NK cells against tumour cells that express high level of activating ligands (Castriconi *et al*, 2004). Worthy of notice, the other transplant settings in this cohort were also reminiscent of those of our previous haploidentical cohort in which the KIR–HLA receptor–ligand model was first established; namely, NK cells were derived from highly purified stem cells (Leung *et al*, 2004); mature T cells and B cells were extensively depleted to provide a lymphopenic environment (Prlic *et al*, 2003; Jamieson *et al*, 2004); and there was no interference by graft-versus-host disease or its treatment (Lowe *et al*, 2003; Cooley *et al*, 2005). All of these factors may contribute significantly to the prominent NK cell effects observed in this study.

The extension of the applicability of the KIR–HLA receptor–ligand model to autologous HCTs is clinically significant, as the number of autologous HCTs performed worldwide annually is twice that of allogeneic HCTs (Center for International Blood and Marrow Transplant Research (CIBMTR) website, 2006). Haploidentical HCTs are performed only in a few centres, whereas autologous HCTs are offered in almost all the transplantation centres. Herein, we demonstrated for the first time that patients with inhibitory KIR–HLA mismatch are at low risk of relapse after autologous HCT. Other novel observation is that the receptor–ligand model may be applicable not only to patients with leukaemia (Leung *et al*, 2004, Hsu *et al*, 2005), but also to patients with lymphoma or solid tumour. One limitation of this study is that the number of patients was small for each disease category. Our novel findings, however, should stimulate future studies in other centres and in larger cohorts with uniform primary diseases. If confirmed, these results will have significant implications for prognostication and selection of patients for autologous HCT.

## Figures and Tables

**Table 1:** Characteristics of 16 consecutive patients who underwent autologous HCT and were grouped by their numbers of inhibitory KIR–HLA receptor–ligand mismatched pairs

**Disease**	**Indication for HCT**	**Disease status at the time of HCT**	**No. of CD133^+^ cells in the graft ( × 10^6^ kg^−1^)**	**Natural cytotoxicity** **(%)^a^**	**HLA-Bw**	**HLA-C**	**KIR mismatch^a^**	**Outcome**
NB	Stage 4	VGPR	7.9	32.0	6/6	Asn80/Asn80	2DL1, 3DL1	Alive, NED
HD	Relapse	PR	5.5	13.1	6/6	Asn80/Asn80	2DL1, 3DL1	Alive, NED
HD	Relapse	CR	4.8	35.0	6/6	Asn80/Asn80	2DL1, 3DL1	Alive, NED
HD	Relapse	PR	5.0	16.8	6/6	Asn80/Asn80	2DL1, 3DL1	Alive, NED
NB	Stage 4	VGPR	7.6	13.5	4/6	Asn80/Asn80	2DL1	Alive, NED
NHL	Relapse	PR	2.1	ND	6/6	Asn80/Lys80	3DL1	Alive, NED
NHL	Relapse	PR	2.2	31.0	6/6	Asn80/Lys80	3DL1	Alive, NED
NB	Stage 4	VGPR	8.0	15.9	6/6	Asn80/Asn80	3DL1	Died of PD
NHL	Relapse	PR	5.9	15.6	4/6	Lys80/Lys80	2DL3	Died of PD
PNET	Metastasis	CR	5.3	16.5	4/6	Asn80/Asn80	2DL1	Died of PD
DSRCT	Metastasis	CR	5.4	18.4	4/6	Asn80/Lys80	None	Alive, NED
NHL	Relapse	PR	2.5	ND	4/6	Asn80/Lys80	None	Died of PD
ES	Relapse	PR	3.8	26.8	4/6	Asn80/Lys80	None	Died of PD
NB	Stage 4	VGPR	7.8	17.7	4/4	Asn80/Lys80	None	Died of PD
NB	Stage 4	PR	6.0	ND	6/6	Asn80/Lys80	None	Died of PD
NB	Stage 4	VGPR	8.0	15.0	4/6	Asn80/Lys80	None	Died of PD

Abbreviations: CR=complete response; DSRBT=desmoplastic small round cell tumour; ES=Ewing's sarcoma; HD=Hodgkin's disease; NB=neuroblastoma; ND=not done; NED=no evidence of disease; NHL=non-Hodgkin's lymphoma; PD=progressive disease; PNET=primitive neuroectodermal tumour; PR=partial response; VGPR=very good partial response.

a Natural cytotoxicity indicates the specific lysis of K562 cells by blood mononuclear cells post-HCT and is used for the analysis of the cytotoxicity model; KIR–HLA mismatch is determined by using the receptor–ligand model.

**Table 2:** Risk factor analysis for event-free survival

**Factor**	**Hazard ratio**	**95% CI**	***P*-value**
Receptor–ligand model (match *vs* mismatch)	5.5	1.3–24.2	0.01
Disease (lymphoma *vs* other solid tumours)	0.9	0.1–7.9	0.94
Disease status (PR *vs* CR/VGPR)	1.3	0.3–5.3	0.71
Cytotoxicity model	0.9	0.7–1.1	0.42
Number of CD133^+^ cells in graft	1.2	0.8–1.8	0.43

Abbreviations: CR=complete response; PR=partial response; VGPR=very good partial response.
